# P2X7 Receptor Antagonist A740003 Mitigates Cardiac Senescence by Inhibiting Inflammaging Through the NF-κB Pathway

**DOI:** 10.3390/ph19101548

**Published:** 2026-09-30

**Authors:** Anfal F. Bin Dayel, Asma S. Alonazi, Alaa Z. Alhajjari, Danah A. Alwakail, Maha A. Alamin, Haya M. Alrajeh, Alaa Alnoor Alameen, Doaa M. Elnagar, Nouf M. Alrasheed

**Affiliations:** 1 Department of Pharmacology and Toxicology, College of Pharmacy, King Saud University, Riyadh 11451, Saudi Arabia; aaloneazi@ksu.edu.sa (A.S.A.); 442200247@student.ksu.edu.sa (A.Z.A.); 442200746@student.ksu.edu.sa (D.A.A.); mahaali@ksu.edu.sa (M.A.A.); hmalrajeh@ksu.edu.sa (H.M.A.); aalameen1.c@ksu.edu.sa (A.A.A.); nrasheed@ksu.edu.sa (N.M.A.); 2 Department of Zoology, College of Science, King Saud University, Riyadh 11451, Saudi Arabia; elnagard1@yahoo.com

**Keywords:** cardiac aging, inflammaging, cardiovascular diseases, A740003, purinergic P2X7 receptors, nuclear factor kappa B, senescence-associated secretory phenotype

## Abstract

**Background:** Inflammaging is a hallmark of cardiac aging that can develop through the NF-κB pathway. Purinergic P2X7 receptors (P2X7Rs) mediate inflammation in cardiovascular diseases. However, the role of P2X7Rs in cardiac inflammaging remains unclear. This research aims to explore the association between P2X7Rs and cardiac inflammaging and whether pharmacological inhibition of P2X7Rs (A740003) can alleviate aging-related cardiac inflammation by regulating the NF-κB pathway in an animal model of cardiac aging. **Methods:** Wistar albino rats were subjected to intraperitoneal administration of D-galactose (200 mg/kg/day) for 10 weeks to induce cardiac aging. Four groups of eight rats each were divided into: normal control (saline for 10 weeks); aging control (D-galactose + saline from weeks 8–10); aging + vehicle (D-galactose + vehicle from weeks 8–10); and aging + A740003 (D-galactose + A740003 (19 mg/kg/day) from weeks 8–10). Heart tissues were then histologically examined and cardiac cellular senescence markers, including SA-β-Gal activity, p16^Ink4a^ and p21^Cip/Waf^ expression, and pro-inflammatory senescence-associated secretory phenotype (SASP) factors, were assessed. Expression of P2X7R, NF-κBp105, and NF-κBp65 was detected. **Results:** Treatment with A740003 reduced cellular senescence markers and pro-inflammatory SASP factors. The expression of P2X7R, NF-κBp105, and NF-κBp65 was significantly increased in cardiac tissues of the aging group, whereas the expression of IκB was decreased. A740003 treatment decreased the levels of P2X7R, NF-κBp105 and NF-κBp65. **Conclusions:** Data indicate that A740003 alleviates cardiac inflammaging through inhibition of the P2X7R-mediated NF-κB mechanism. Therefore, the P2X7R-mediated NF-κB pathway may provide a novel strategy for mitigating age-related inflammation and preventing age-related cardiovascular diseases.

## 1. Introduction

Cardiovascular diseases still represent a serious health challenge around the world, causing a significant number of deaths each year. The incidence of cardiovascular disease is strongly linked to aging, with an incidence rate of approximately 75% among people aged 60 to 79 years old and more than 85% among those aged 80 and older [1]. In Saudi Arabia, the prevalence of cardiovascular disease is ~1.2% for people aged 40 and under, ~6.5% for people aged 60 to 64, and ~11% for those aged 65 and over [2]. This strong link between aging and the prevalence of cardiovascular disease highlights the importance of understanding the cellular and molecular mechanisms of cardiac aging in order to identify potential approaches to preventing and/or delaying age-related cardiovascular disease.

Cardiac aging is the structural and functional deterioration of the cardiovascular system that occurs with advancing age. These are normal physiological changes, but they raise the risk of cardiovascular disease [3]. Senescent cardiac cells are associated with several key characteristics. At the morphological level, senescent cardiac cells enlarge and become flattened and exhibit lysosomal expansion which is indicated by elevated senescence-associated β-galactosidase (SA-β-Gal) [4,5,6]. At the cellular level, the proliferative capacity of cells declines with age and is distinguished by the cessation of the cell cycle in the G1 and G2 phases, resulting in a permanent loss of proliferative capacity [7]. The activation of cyclin-dependent kinase inhibitors, such as p16^Ink4a^ and p21^Cip/Waf^ [8,9], indicates irreversible cell cycle arrest in senescent cells. At the immune level, aging causes immune systems to become dysregulated, resulting in insufficient senescent cell clearance. These senescent cells accumulate and produce the senescence-associated secretory phenotype (SASP), which includes pro-inflammatory cytokines such as tumor necrosis factor-alpha (TNF-α), and interleukin (IL)-6, as well as growth factors, proteases, and chemokines [10]. SASP production can induce a chronic state of low-grade inflammation known as inflammaging [11,12].

Inflammaging is highly linked to the development of cardiovascular disease [13]. Nuclear factor kappa B (NF-κB) is an important mediator of inflammaging processes in the heart [14]. Normally, NF-κB remains as an inactive NF-κBp50/p65 complex in the cytoplasm, which is sequestered by the inhibitory protein IκB. NF-κB p105, a precursor of the p50 subunit, acts similarly to IκB by sequestering NF-κB complexes in the cytoplasm. The p65 subunit has a transcriptional activation domain but remains inactive when bound to IκB [15]. During stimulation, IκB undergoes phosphorylation and degradation, allowing the release of NF-κB. Meanwhile, p105 produces the p50 subunit and forms with p65 the active NF-κBp50/p65 complex. This complex transfers to the nucleus and stimulates the transcription of inflammation-related genes [16,17]. A previous study found that NF-κB activation in aged animal hearts promotes cardiomyocyte senescence and increases the expression of pro-inflammatory SASP cytokines [18]. By contrast, inhibition of Ca^2+^/calmodulin-dependent protein kinase II reduced NF-κB activation and expression of these pro-inflammatory SASP factors, thereby attenuating aging-associated cardiac inflammation [18].

Purinergic receptors, particularly P2X7 receptors (P2X7Rs), play an important role in cardiac diseases [19]. P2X7R is an ATP-gated ion channel that remains inactive in physiological conditions due to low extracellular ATP concentrations. However, when the extracellular ATP concentration rises under stress and pathological conditions, P2X7R becomes active, leading to increased permeability for many cations such as K^+^, Na^+^, and Ca^2+^ and activation of various cellular signaling pathways [20,21]. P2X7R regulates cardiac mitochondrial function, and its absence is associated with reduced mitochondrial respiration and ATP production, as well as a decline in cardiac performance [22]. Interestingly, P2X7R is observed to participate in cardiac aging regulation. A prior study showed that P2X7R levels in humans and animals increase with age, and deleting P2X7R mitigates cardiac senescence by promoting mitochondrial autophagy [23]. This correlation between P2X7R and cardiac aging requires further studies to elucidate the role of P2X7R in other aspects of cardiac aging, such as cardiac inflammaging.

P2X7Rs regulate inflammation through the NF-κB pathway [24]. Several cardiac diseases have been linked to inflammation through the P2X7R/NF-κB pathway. For example, activation of the P2X7R in myocardial ischemia leads to increased NF-κB activity and the release of pro-inflammatory cytokines [25]. Conversely, inhibiting P2X7Rs with short hairpin RNA can reduce post-myocardial infarction inflammation by suppressing NF-κB activity [26]. P2X7Rs also regulate inflammation by acting on the NLRP3 inflammasome, which can activate caspase-1 and process pro-IL-1β and pro-IL-18 to their active form [27]. The P2X7R/NLRP3 pathway has been linked to inflammation in several cardiac diseases. For example, activation of the P2X7R in myocardial infarction triggers NLRP3 inflammasome activity and IL-1β release [28]. In contrast, a pharmacological P2X7R antagonist can prevent the formation of an inflammasome and reduce the size of the infarct after a myocardial infarction [29]. A740003, a selective pharmacological inhibitor of P2X7R with low activity on other P2X and P2Y receptor subtypes [30], is a P2X7R antagonist with beneficial cardiovascular effects in several experimental models of inflammation-related cardiovascular disease. For example, A740003 was shown to mitigate maladaptive cardiac remodeling following myocardial infarction by inhibiting the NLRP3-IL-1β inflammatory pathway [28]. Furthermore, A740003 was found to decrease the pro-inflammatory factors IL-1β and MMP-9 in atherosclerotic vascular tissue [31]. It also reduces inflammation in experimental autoimmune myocarditis by suppressing myocardial inflammatory cell infiltration [32]. Collectively, these studies highlight the potential use of A740003 as a possible therapeutic target for treating inflammation associated with cardiovascular disease. Although previous studies have found a link between P2X7R and inflammation-related cardiac diseases, the relationship between P2X7R and cardiac inflammaging remains unknown. Therefore, our aim in this study is to investigate the possible role of P2X7R in cardiac inflammaging and whether the P2X7R antagonist, A740003, has an anti-inflammaging effect by inhibiting the NF-κB signalling pathway in an experimental rat model of cardiac aging. This study expands our understanding of the molecular mechanisms underlying age-related cardiac changes and points to P2X7R as a prospective pharmacological target for mitigating or delaying the progression of age-related cardiac deterioration.

## 2. Results

### 2.1. Effect of A740003 on Heart Weight to Body Weight

The heart weight to body weight (HW/BW) was calculated as an indicator of cardiac hypotrophy in aging rats. As shown in Table 1, no difference was observed in the HW/BW ratio between the normal control rats and aging control rats. This result was observed when aging rats were treated with a drug vehicle and A740003.

### 2.2. A740003 Improves Histopathological Changes and Reduces Senescence-Associated β-Galactosidase in Cardiac Aging Rats

**Figure 1:**
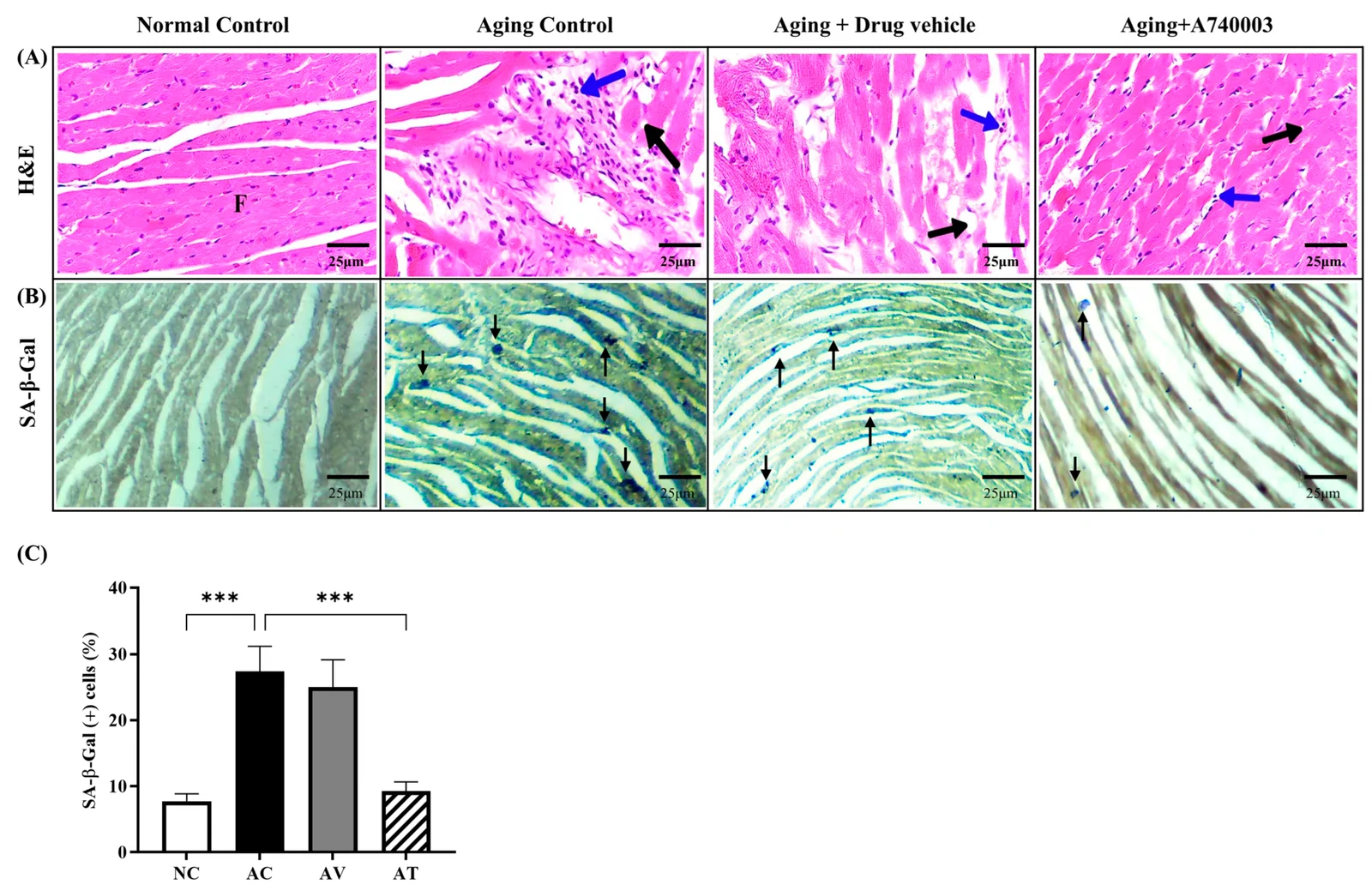
Effects of A740003 on cardiac architecture and senescent cardiac cells. (**A**) Hematoxylin and Eosin (H&E)-stained cardiac tissue shows normal cardiac fiber (F) with no pathological changes in the normal control group, muscle destruction and vacuolar degeneration (black arrow) within cardiac muscle fibers and infiltrative cells (blue arrow) in the aging control group, vacuolar degeneration within cardiac muscle fibers and infiltrative cells in aged rats treated with drug vehicle, and a few infiltrative cells and vacuolar degeneration in aged rats treated with A740003 (magnification 400×). (**B**) Representative senescence-associated β-galactosidase (SA-β-Gal) staining in rat cardiac tissues show no SA-β-Gal staining in the normal control group, increased SA-β-Gal staining in the aging control and aging treated with drug vehicle groups (identified by blue spots of staining around cardiac muscle fibers with black arrows), and marked reduction in SA-β-Gal staining in the aging treated with A740003 group (magnification 40×). (**C**) Percentage of SA-β-Gal-positive cells was quantified using ImageJ software version 1.53t. Values are expressed as mean ± SEM (*n* = 8 different rats/group). One-way analysis of variance (ANOVA) followed by Dunnett’s multiple comparisons test was performed. *** *p* < 0.001 compared to the aging control (AC) group. Abbreviations: NC, normal control; AC, aging control; AV, aging treated with drug vehicle; AT, aging treated with A740003.

Cardiac muscles of normal control rats showed normal structure with no pathological changes. However, cardiac muscles of aging rats exhibited severe alterations manifested by destruction of fibers, vacuolar degeneration of fibers and accumulation of infiltrative cells. Similarly, cardiac muscles of aging rats treated with drug vehicle exhibited severe changes as the aging rats. A740003 treatment improved histopathological changes in cardiac aging rats by restoring normal cardiac structure and reducing vacuolar degeneration and inflammatory cells (Figure 1A). Cardiac aging was identified by the presence of SA-β-Gal in the cardiac tissue. SA-β-Gal blue staining was observed around the cardiac muscle fibers in both the aging control group and the aging group treated with the drug vehicle. In contrast, A740003 treatment revealed reduced SA-β-Gal staining in the aging group (Figure 1B). The SA-β-Gal-positive cells increased from 7.75% ± 1.09 in the normal control group to 27.38% ± 3.79 in the aging control group (*p* < 0.001), representing an increase of 3.53-fold and indicating a significant elevation of senescent cells in cardiac tissues. However, A740003 treatment in aging rats marked ameliorated SA-β-Gal-positive cells about 66% in cardiac tissues compared with the aging control group (*p* < 0.001) (Figure 1C), suggesting that the P2X7R antagonist attenuates cardiac senescent cells.

### 2.3. A740003 Reduces Cyclin-Dependent Kinase Inhibitors in Cardiac Aging Rats

**Figure 2:**
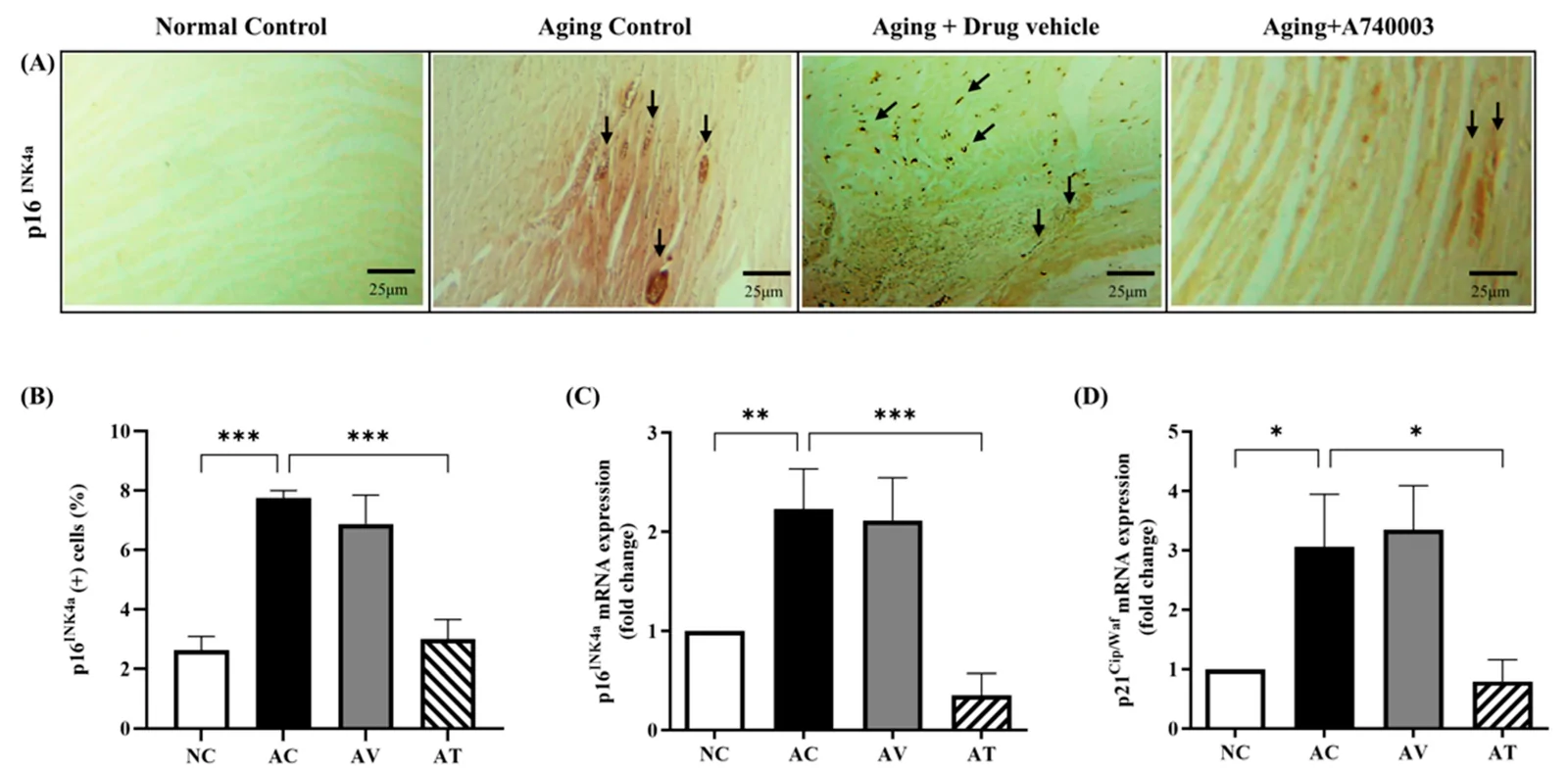
Effect of A740003 on cyclin-dependent kinase inhibitors in cardiac tissues. (**A**) Representative immunohistochemical images of p16^Ink4a^ protein expression in rat heart tissues show no p16^Ink4a^ expression in the normal control group, increased expression in the aging control and aging treated with drug vehicle groups (indicated by dark brown staining with black arrows) and decreased expression in the aging treated with A740003 group (magnification 40×). (**B**) Percentage of p16^Ink4a^ protein expression was quantified using ImageJ software. (**C**) *p16^Ink4a^* and (**D**) *p21^Cip/Waf^* mRNA expression in rat heart tissues. Values are expressed as mean ± SEM (*n* = 5–8 different rats/group). One-way analysis of variance (ANOVA) followed by Dunnett’s multiple comparisons test was performed. * *p* < 0.05, ** *p* < 0.01, *** *p* < 0.001 compared to the aging control (AC) group. Abbreviations: NC, normal control; AC, aging control; AV, aging treated with drug vehicle; AT, aging treated with A740003.

Cyclin-dependent kinase inhibitors p16^INK4a^ and p21^Cip/Waf^ were examined as aging markers in cardiac tissues. Immunohistochemical analysis revealed that p16^INK4a^ protein expression, indicated by brown nuclei and cytoplasmic staining in cardiac tissue, were higher in aging control rats and aging rats treated with a drug vehicle. In contrast, A740003 treatment showed significantly lower p16^INK4a^ protein expression (Figure 2A). Figure 2B depicts the quantification of p16^INK4a^ immunohistochemical results, which show that p16^INK4a^-positive expression was 2.95-fold higher in aging control rats than in normal control rats (*p* < 0.001). On the contrary, aging rats treated with A740003 exhibited a significant 2.58-fold decrease in p16^INK4a^ expression compared to aging control rats (*p* < 0.001). Following the evaluation of protein expression, the mRNA expressions of *p16^INK4a^* and *p21^Cip/Waf^* were examined to identify changes at the gene transcription level. The expression of *p16^INK4a^* and *p21^Cip/Waf^* genes was significantly increased in aging control rats (*p* < 0.01 and *p* < 0.05, respectively) and decreased in A740003-treated aging rats (*p* < 0.001 and *p* < 0.05, respectively) (Figure 2C,D).

### 2.4. A740003 Reduces Inflammaging Markers in Serum and Cardiac Tissue Rats

**Figure 3:**
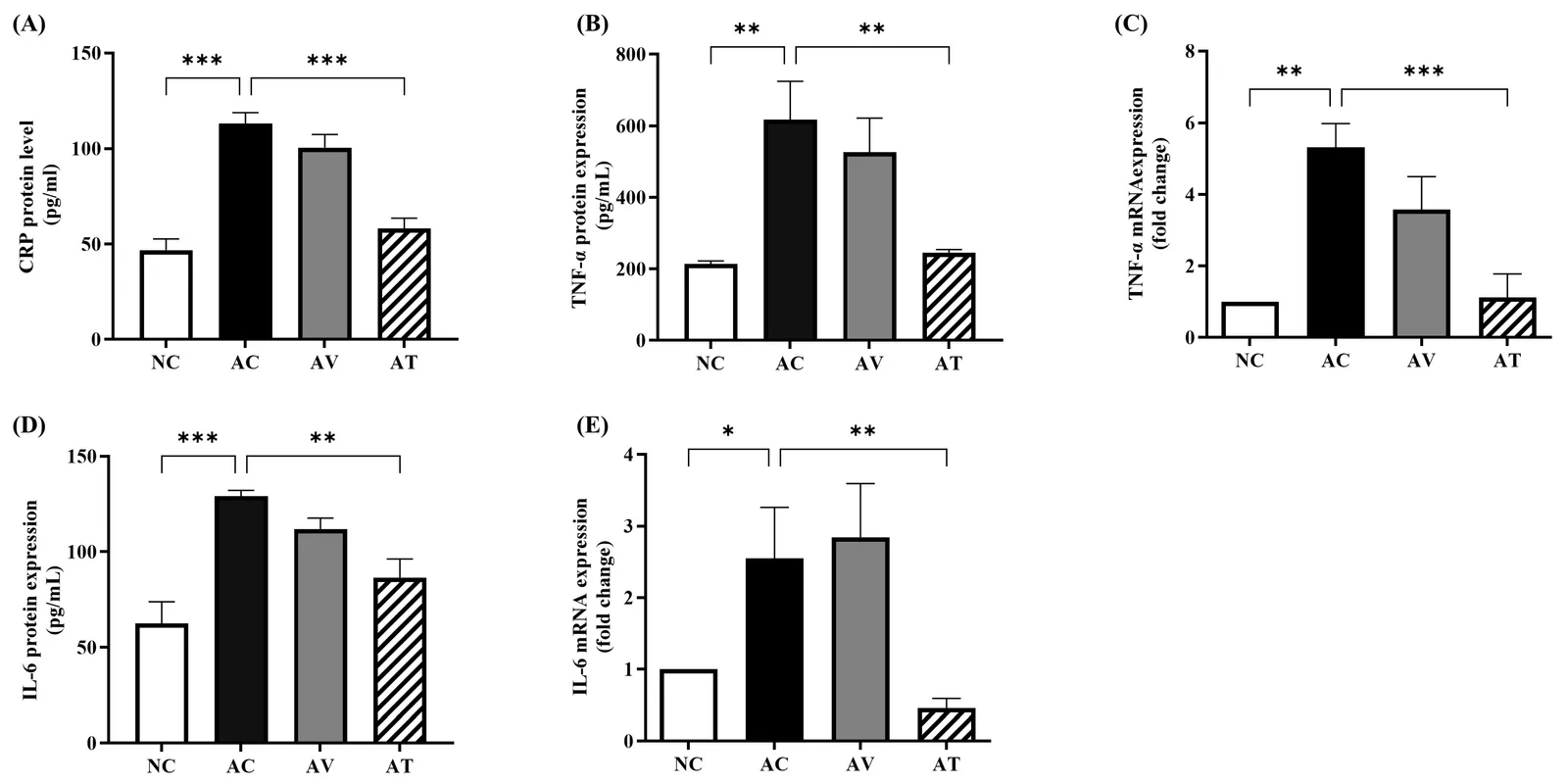
Effect of A740003 on inflammaging markers in serum and cardiac tissue. (**A**) CRP levels in serum. (**B**) TNF-α protein expression and (**C**) *TNF-α* gene expression in heart tissues. (**D**) IL-6 protein expression and (**E**) *IL-6* gene expression in heart tissues. Values are expressed as mean ± SEM (*n* = 5–8 different rats/group). One-way analysis of variance (ANOVA) followed by Dunnett’s multiple comparisons test was performed. * *p* < 0.05, ** *p* < 0.01, *** *p* < 0.001 compared to the aging control (AC) group. Abbreviations: CRP, C-reactive protein; TNF-α, tumor necrosis factor-alpha; IL-6, interleukin-6; NC, normal control; AC, aging control; AV, aging treated with drug vehicle; AT, aging treated with A740003.

CRP was measured in serum as a systemic inflammation marker. As shown in Figure 3A, the CRP was significantly increased by ~2.4-fold in the aging control rats compared to that in the normal control rats (*p* < 0.001), whereas treatment of aging rats with A740003 significantly reduced CRP by ~1.95-fold compared with aging control rats (*p* < 0.001). In addition, SASP, including TNF-α and IL-6, was assessed as an inflammaging marker in cardiac tissues. Initially, we measured the protein expression of TNF-α and IL-6 proteins and then the mRNA expression in cardiac tissue to determine whether the observed changes seen in protein expression were consistent with the changes in gene transcription. ELISA results showed that the protein expressions of TNF-α and IL-6 were significantly increased by ~2.8-fold and ~2.1-fold in the aging control rats compared to normal control rats (*p* < 0.01 and *p* < 0.001, respectively). Treatment of aging rats with A740003 significantly reduced TNF-α and IL-6 protein expressions by ~2.5-fold and 1.5-fold, respectively, as compared with aging control rats (*p* < 0.01) (Figure 3B,D). According to RT-PCR results, *TNF-α* and *IL-6* mRNA expressions were significantly increased by ~5.3-fold and 2.5-fold in the aging control rats compared to normal control rats (*p* < 0.01 and *p* < 0.05, respectively). Treatment of aging rats with A740003 significantly reduced *TNF-α* and *IL-6* mRNA expressions by ~4.7-fold and 5.6-fold as compared with aging control rats (*p* < 0.001 and *p* < 0.01, respectively) (Figure 3C,E).

### 2.5. A740003 Reduces P2X7R Expression in Cardiac Inflammaging Rats

**Figure 4:**
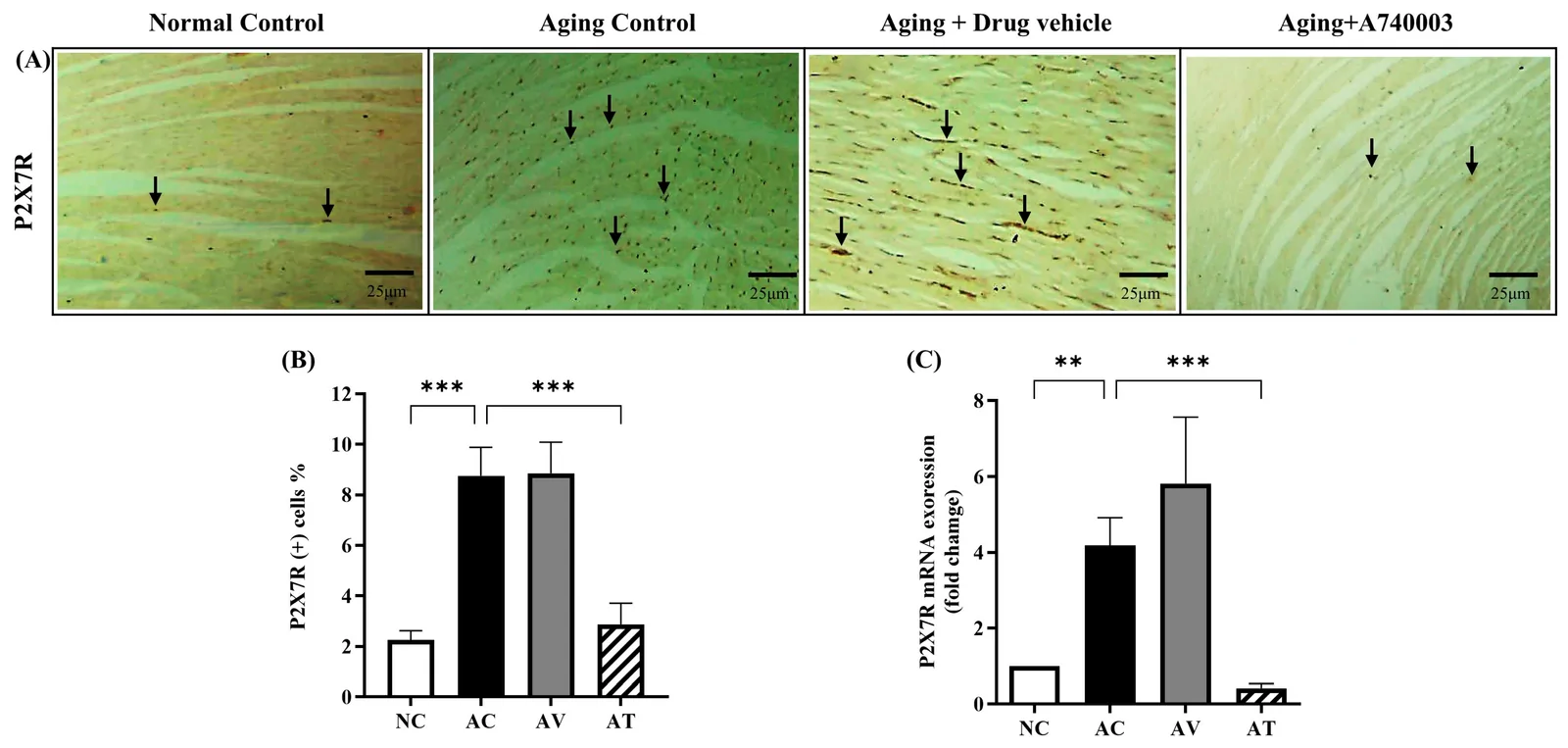
Effect of A740003 on P2X7R expression in cardiac tissues. (**A**) Representative immunohistochemical images of P2X7R expression in rat heart tissues show normal P2X7R expression in the normal control group, increased expression in the aging control and aging treated with drug vehicle groups (indicated by brown spots of staining with black arrows) and decreased expression in the aging treated with A740003 group (magnification 40×). (**B**) Percentage of P2X7R expression was quantified using ImageJ software. (**C**) *P2X7R* mRNA expression in rat heart tissues. Values are expressed as mean ± SEM (*n* = 5–8 different rats/group). One-way analysis of variance (ANOVA) followed by Dunnett’s multiple comparisons test was performed. ** *p* < 0.01, *** *p* < 0.001 compared to the aging control (AC) group. Abbreviations: NC, normal control; AC, aging control; AV, aging treated with drug vehicle; AT, aging treated with A740003.

Immunohistochemical analysis revealed that P2X7R protein expression, indicated by brown scattered staining in cardiac tissue, were higher in aging control rats and aging rats treated with a drug vehicle. In contrast, A740003 treatment showed significantly lower P2X7R protein expression (Figure 4A). Figure 4B depicts the quantification of P2X7R immunohistochemical results, which show that P2X7R-positive expression was 3.88-fold higher in aging control rats than in normal control rats (*p* < 0.001). On the contrary, aging rats treated with A740003 exhibited a significant 3-fold decrease in P2X7R expression compared to aging control rats (*p* < 0.001). To determine whether high P2X7R protein expression is associated with changes in gene transcription, mRNA expression was measured. According to RT-PCR results, *P2X7R* mRNA expression was significantly increased by ~4-fold in the aging control rats compared to normal control rats (*p* < 0.01). Treatment of aging rats with A740003 significantly reduced *P2X7R* mRNA expression by 5.8-fold as compared with aging control rats (*p* < 0.001) (Figure 4C).

### 2.6. A740003 Regulates NF-κB/IκB Expression in Cardiac Inflammaging Rats

**Figure 5:**
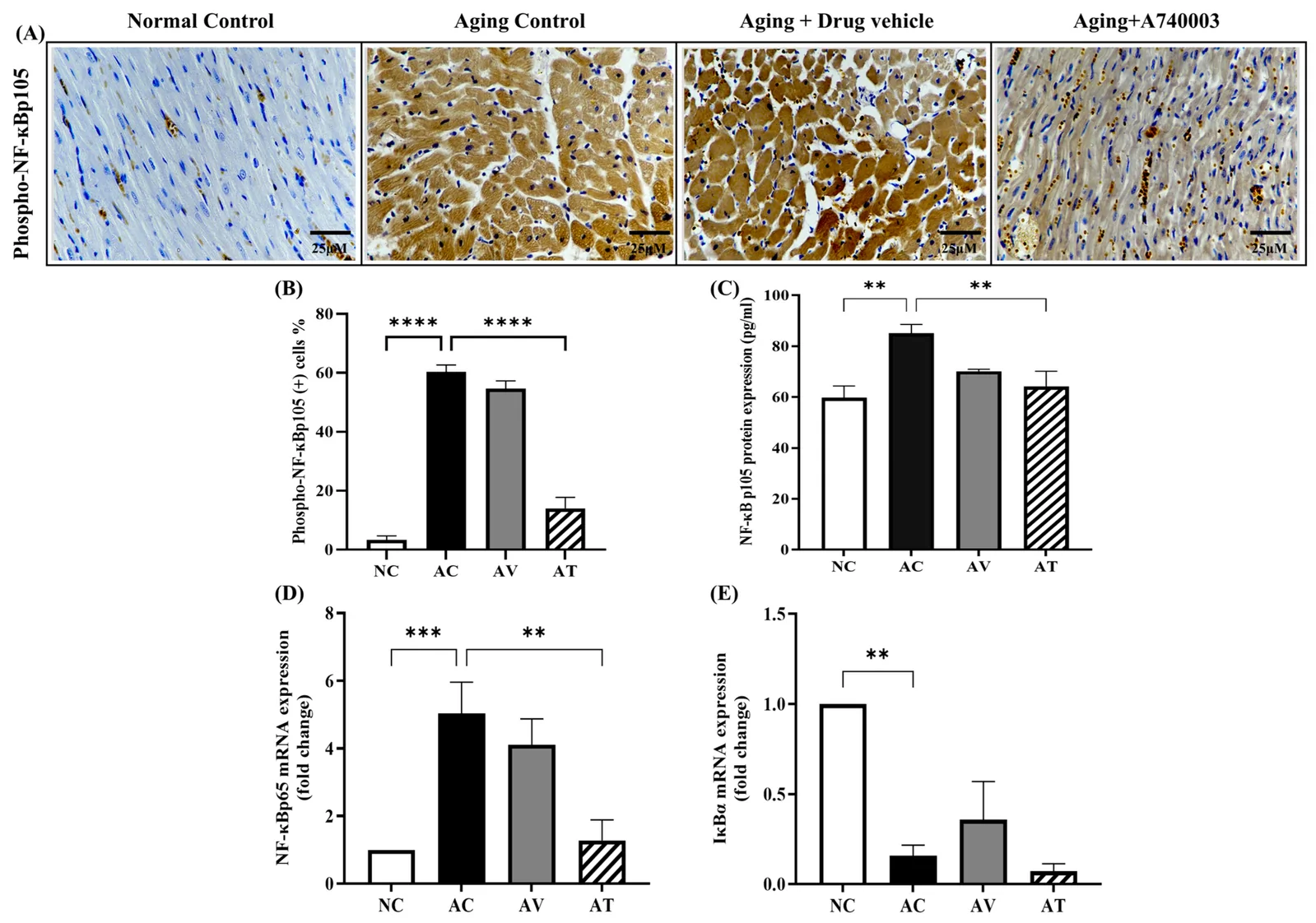
Effect of A740003 on NF-κB/IκB expression in cardiac tissues. (**A**) Representative immunohistochemical images of phospho-NF-κBp105 expression in rat heart tissues reveal weak phosphor-NF-κBp105 expression in the normal control group, increased expression in the aging control and aging treated with drug vehicle groups (dark brown staining) and decreased expression in the aging treated with A740003 group (magnification 400×). (**B**) Percentage of phospho-NF-κBp105 expression was quantified using ImageJ software. Values are expressed as mean ± SEM (*n* = 3 different rats/group). (**C**) NF-κBp105 protein expression in cardiac tissues. (**D**) *NF-κBp65* and (**E**) *IκBα* gene expression in cardiac tissues. Values are expressed as mean ± SEM (*n* = 5–8 different rats/group). One-way analysis of variance (ANOVA) followed by Dunnett’s multiple comparisons test was performed. ** *p* < 0.01, *** *p* < 0.001, **** *p* < 0.0001 compared to the aging control (AC) group. Abbreviations: NC, normal control; AC, aging control; AV, aging treated with drug vehicle; AT, aging treated with A740003.

Immunohistochemistry analysis showed that phospho-NF-κBp105 expression was upregulated in the cardiac tissue of aging rats, while treatment with A740003 showed phospho-NF-κBp105 expression was downregulated (Figure 5A). The quantification of phospho-NF-κBp105 immunohistochemical results in Figure 5B shows that phospho-NF-κBp105-positive expression was 18-fold higher in aging control rats than normal control rats (*p* < 0.0001). However, significant amelioration of phospho-NF-κBp105-positive cells about 4.3-fold in cardiac tissues was observed in aging rats treated with A740003 compared with the aging control group (*p* < 0.0001). ELISA results showed that NF-κBp105 protein expression was significantly increased by 42.3% in the cardiac tissue of aging control rats compared to that in the normal control rats (*p* < 0.01), whereas treatment of aging rats with A740003 significantly reduced NF-κBp105 protein expression by 32.5% compared with aging control rats (*p* < 0.01) (Figure 5C). RT-PCR analysis showed that *NF-κBp65* gene expression was significantly increased by ~5-fold in the cardiac tissue of aging control rats compared to normal control rats (*p* < 0.001). Treatment of aging rats with A740003 significantly reduced *NF-κBp65* mRNA expression by ~4-fold, as compared with aging control rats (*p* < 0.01) (Figure 5D). *IκBα* gene expression was significantly decreased by 84.2% in the cardiac tissue of aging control rats compared to normal control rats (*p* < 0.01) (Figure 5E).

## 3. Discussion

Cardiac aging is a decline in the function and structure of the cardiovascular system that predisposes to the risk of cardiovascular disease and death, making it a key factor in determining life expectancy. In this study, we developed a model of cardiac aging in Wistar albino rats by injecting them with D-galactose, a reducing monosaccharide. This model has been shown to accelerate the aging process in the heart, triggering cellular and molecular alterations in cardiac tissue [33,34]. Similar to a previous study [35], our findings showed that rats treated with D-galactose had no change in body weight, heart weight, or HW/BW%. However, other studies have reported that D-galactose administration increases heart weight indices [33]. Such variations could be explained by differences in animal age, strain, drug dose, treatment duration, and other experimental parameters. Notably, the absence of cardiac hypertrophy does not rule out D-galactose-induced cardiac aging, as D-galactose can cause a variety of other cardiac aging features, such as inflammation, mitochondrial dysfunction, and structural changes [33], which can occur without changes in heart weight indices. Consistent with previous studies [36,37], our findings revealed that rats treated with D-galactose exhibited histopathological changes in the heart, including fiber destruction, vacuolar fiber degeneration, and accumulation of infiltrating cells. Pathological cardiac remodeling is most likely caused by galactose accumulation in cardiac cells, which promotes reactive oxygen species, oxidative stress, advanced glycation products, and inflammation [33]. The successful establishment of the cardiac aging model was confirmed by the detection of cellular senescence markers, including SA-β-Gal and cyclin-dependent kinase inhibitors (p16^Ink4a^ and p21^Cip/Waf^). Our results showed that D-galactose treatment increased SA-β-Gal activity and expression of cyclin-dependent kinase inhibitors, consistent with previous studies [36,37,38,39]. The increase in SA-β-gal-positive cells observed in the cardiac tissue of aging rats can be attributed to the accumulation of senescent cells characterized by high β-galactosidase activity in the lysosome [5]. Senescent cardiomyocyte was confirmed by promoting cell cycle arrest through up-regulated expression of p16^Ink4a^ and p21^Cip/Waf^ in the cardiac tissue of aging rats. These data indicate the successful establishment of cardiac aging in an animal model.

Inflammaging is a feature of cardiac aging that can contribute to the development and progression of cardiovascular disease [11,13]. Systemic CRP levels and cardiac SASP factors TNF-α and IL-6 have been used as biomarkers for inflammaging. CRP expression reportedly increases in cardiac aging mice [40]. Our findings indicate that D-galactose treatment in rats increased systemic CRP levels, which is consistent with a previous study [41]. Furthermore, previous research has shown that D-galactose stimulates SASP through mitochondrial dysfunction and oxidative damage, promoting senescent cells to produce pro-inflammatory cytokines [33,42]. Consistently, our results showed that D-galactose treatment rats had higher cardiac SASP factors of TNF-α and IL-6, indicating the accumulation of senescent cells and their pro-inflammatory cytokines during the process of cardiac aging. These results confirm the successful induction of cardiac inflammatory activity in an aged animal model. To fully assess the inflammatory response, additional pro-inflammatory cytokines such as IL-1β, IL-18, IFN-γ, and IL-8 should be evaluated.

P2X7R reportedly plays a significant regulatory role in cardiac aging, with high levels of P2X7R observed in mice with cardiac aging and in humans with advancing age [23]. Our results consistently showed that D-galactose-treated rats exhibited a marked increase in P2X7R expression. This upregulation of P2X7R in cardiac aging could be explained by D-galactose-induced oxidative stress and mitochondrial dysfunction [33,42], which can cause cellular stress and ATP release to extracellular space, and subsequently activate P2X7Rs [43]. The present study showed that increased P2X7R expression in the cardiac tissue was accompanied by cardiac structural abnormalities and senescence of cardiomyocytes with increased SA-β-gal activity and upregulated p16^Ink4a^ and p21^Cip/Waf^ expression. A previous study showed that P2X7R deletion (P2X7R^−^/^−^) in old mice attenuated cardiac dysfunction and cellular senescence markers [23]. Consistently, our results revealed that the P2X7R antagonist A740003 attenuated cardiac aging features in D-galactose-treated rats by restoring normal cardiac structure and decreasing cellular senescence markers, including SA-β-Gal, p16^Ink4a^ and p21^Cip/Waf^. These findings suggest that P2X7R plays a role in age-related cardiac structural abnormalities and cellular senescence.

**Figure 6:**
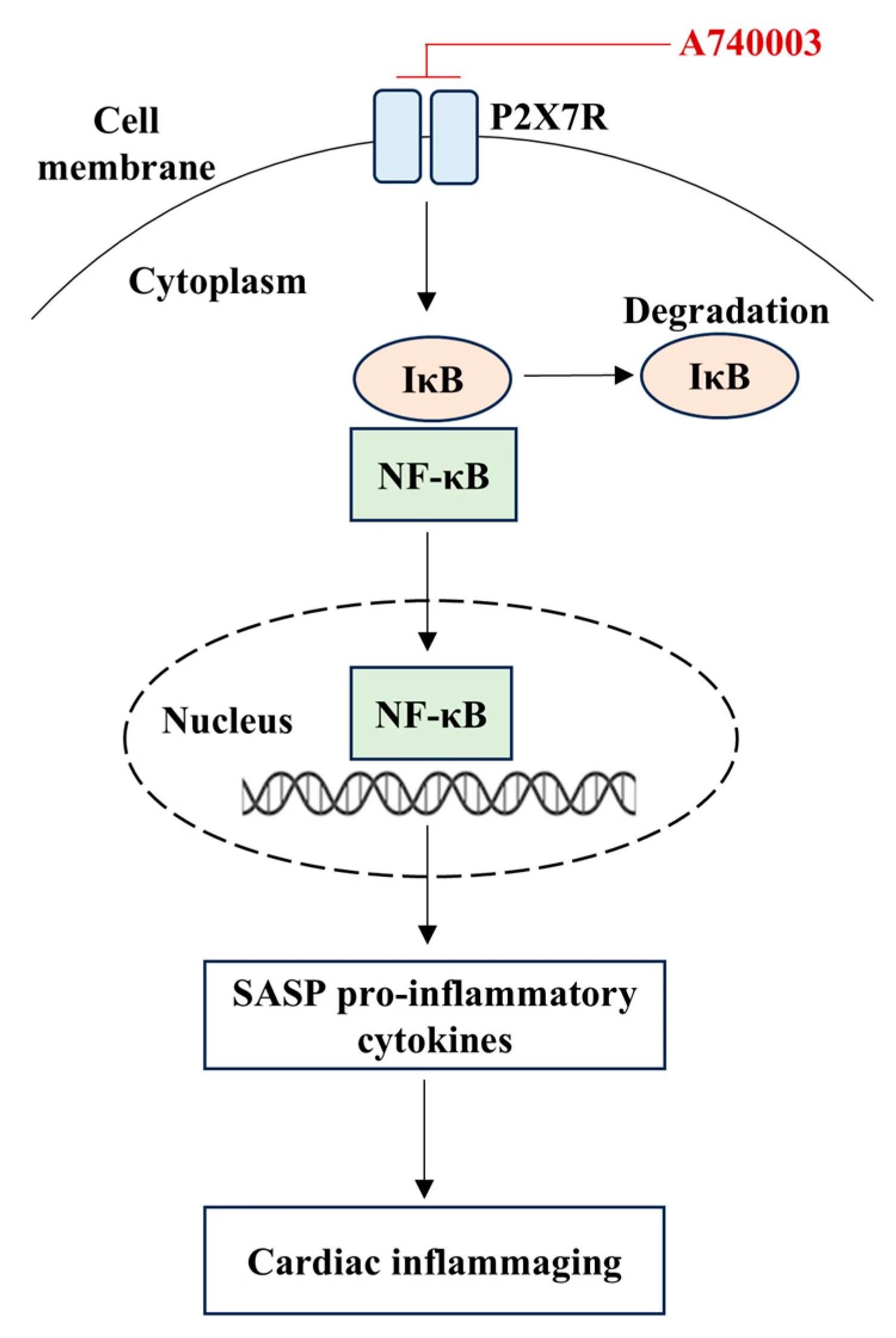
Diagrammatic illustration of the mechanism by which P2X7R antagonist A740003 alleviates cardiac inflammaging by inhibiting the NF-κB pathway. Abbreviations: IκB, inhibitor of κB; NF-κB, nuclear factor κB; P2X7R, P2X7 receptors; SASP, senescence-associated secretory phenotype.

P2X7Rs mediate inflammation in cardiovascular diseases by regulation of the NF-κB pathway [19,44]. However, it is unclear whether P2X7Rs contribute to inflammation associated with cardiac aging. Therefore, we aimed to examine whether P2X7Rs mediate cardiac inflammaging and reveal the underlying mechanisms. Earlier studies have demonstrated that overexpression of P2X7Rs in cardiovascular diseases increases NF-κB activity [19,44]. Similarly, our results showed that overexpression of P2X7Rs in cardiac aging was associated with upregulation of the NF-κB pathway, as evidenced by increased levels of NF-κBp105 phosphorylation and its protein, along with increased *NF-κBp65* mRNA and decreased *IκBα* mRNA. High levels of NF-κB p105 protein and phosphorylation indicate an increased availability of the NF-κB precursor, while high levels of *NF-κBp65* mRNA indicate an increased synthesis of the active NF-κB subunit. Meanwhile, low *IκBα* mRNA levels indicate reduced inhibition of NF-κB, which promotes its activation and translocation into the nucleus [16,17]. Collectively, these results suggest that cardiac aging stimulates the P2X7R-NF-κB pathway, which is characterized by increased expression of P2X7R and NF-κB subunits with decreased IκB-mediated inhibition. The upregulation of the P2X7R-mediated NF-κB pathway in aging cardiac tissue was associated with elevated systemic CRP levels and cardiac SASP factors TNF-α and IL-6, suggesting that P2X7Rs mediate cardiac inflammaging by regulating the NF-κB pathway. This is consistent with previous cardiovascular disease studies, which have shown that activation of P2X7R is associated with increased pro-inflammatory cytokines, including TNF-α and IL-6, with a positive correlation between P2X7R and systemic CRP levels in ischemic and myocardial infarction states [44,45,46]. The involvement of P2X7Rs in cardiac inflammaging was verified by use of the P2X7R antagonist A740003. Our results showed that P2X7R antagonist A740003 reduced the phosphorylation levels of NF-κBp105 and its protein and reduced *NF-κBp65* mRNA, indicating that P2X7R antagonist A740003 inhibits both NF-κB expression and activation. Consequently, NF-κB becomes less able to stimulate the transcription of the *IκBα* gene, and this may explain the lack of effect of the P2X7R antagonist A740003 on the expression of *IκBα* mRNA. Interestingly, downregulation of the P2X7R-mediated NF-κB pathway in aging cardiac tissue was associated with decreased systemic CRP levels and cardiac SASP factors TNF-α and IL-6. These data illustrate that the P2X7R antagonist A740003 alleviates cardiac inflammaging by inhibiting the NF-κB pathway (Figure 6). Mechanistically, P2X7R activation can promote NLRP3 inflammasome assembly and subsequent caspase-1 activation leading to the maturation and release of the pro-inflammatory cytokines IL-1β and IL-18. Thus, inhibition of P2X7R by A740003 may potentially attenuate cardiac inflammaging not only through modulation of NF-κB signaling but also through suppression of NLRP3 inflammasome-mediated inflammatory response [44,47]. To our knowledge, this is the first direct evidence to show that the P2X7R antagonist A740003 alleviates aging-related cardiac inflammation through the NF-κB pathway, highlighting that P2X7R is a potential therapeutic target for alleviating age-related cardiac deterioration and preventing the development of age-associated cardiovascular disease.

### Study Limitations and Future Research

Certain limitations of this research should be noted. First, the effect of P2X7R antagonist A740003 in non-aged hearts was not investigated; thus, further evaluation is needed to provide essential information under normal cardiac physiological conditions. Second, cardiac function assessments were not included in the study; therefore, further evaluation is required to confirm the efficacy of A740003 using electrocardiography, echocardiography and blood pressure measurement. Third, this study demonstrates the involvement of the NF-κB signaling pathway but does not directly assess its nuclear localization. Further studies based on protein analysis techniques—such as nuclear/cytoplasmic fractionation or immunohistochemical/immunofluorescence analysis specifically evaluating nuclear p65 localization—are needed to provide direct evidence of NF-κB activation. Fourth, this study did not investigate the biological effects of P2X7R agonists; such research could provide complementary mechanistic evidence regarding the involvement of P2X7R in cardiac aging and require further investigation. Fifth, the use of a single A740003 dose in this study limits the assessment of dose-dependent effects, which should be addressed in future studies. Finally, this study lacks genetically modified mouse models, indicating the need for future studies using such models to provide further evidence of the causal role of P2X7R in cardiac aging and to elucidate the underlying molecular mechanisms. Future studies addressing these limitations would enhance and reinforce the interpretation of the current findings.

## 4. Materials and Methods

### 4.1. Drugs, ELISA Kits, and Antibodies

A740003 (HY-50697) was obtained from MedChemExpress (Monmouth Junction, NJ, USA) and D-galactose (03900) was procured from Lobe Chemie Pvt. Ltd. (Mumbai, India). SA-β-Gal stain (G1580) was obtained from Solarbio Life Science (Beijing, China). Rat-specific enzyme-linked immunosorbent assay (ELISA) kits for TNF-α (SEKR-0009), C-reactive protein (CRP) (SEKR-0017), IL-6 (SEKR-0005) and NF-κBp105 (SEKR-0148) were procured from Solarbio Life Science (Beijing, China). Primary antibodies, including the anti-P2X7 polyclonal antibody (K011675P) and the anti-CDKN2A/p16INK4a polyclonal antibody (K010139P), were obtained from Solarbio Life Science (Beijing, China). Primary antibody anti-NF-κBp105 (phospho Ser932) (ES1367) was purchased from ELK Biotechnology (Wuhan, China). Mouse and rabbit conjugated secondary antibodies were supplied by Sigma-Aldrich (St. Louis, MO, USA).

### 4.2. Animals

The Animal Care Center of the College of Pharmacy at King Saud University (KSU) provided thirty-two male Wistar albino rats aged six weeks and weighing 130 to 150 g. Rats were kept in an ideal environment, with an air-conditioned room (25 °C), 60% humidity, and an unlimited supply of water and food. The study was approved by the Research Ethics Committee at KSU [Ethical Reference Number: KSU-SE-25-1 (28 January 2025)], and laboratory animals were used in compliance with the rules of the Animal Care Center of KSU.

### 4.3. Experimental Animals

**Figure 7:**
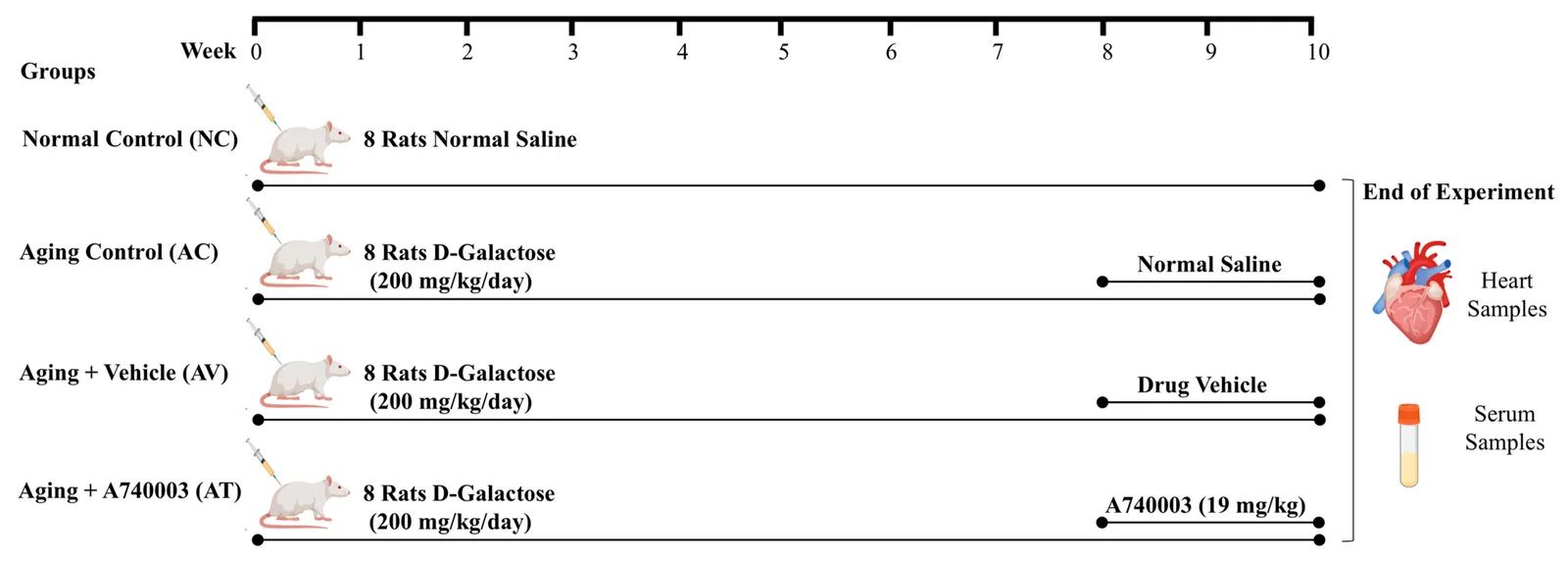
Design and treatment of experimental animals.

Four groups of eight rats each were formed (Figure 7). Cardiac aging was induced by intraperitoneal injection with D-galactose (200 mg/kg/day) dissolved in distilled water for 10 weeks [33]. The rats in the control group were given normal saline, and the other groups were treated with a drug vehicle or A740003 based on the following: Normal Control (NC) Group: rats were injected intraperitoneally with normal saline (0.9% NaCl) daily for 10 weeks; Aging Control (AC) Group: rats were injected intraperitoneally with D-galactose (200 mg/kg/day) dissolved in distilled water for 10 weeks [33], and normal saline (0.9% NaCl) was administered intraperitoneally daily from week 8 to 10; Aging + Drug Vehicle (AV) Group: rats were injected intraperitoneally with D-galactose (200 mg/kg/day) dissolved in distilled water for 10 weeks [33], and the drug vehicle (2 mL/kg) was administered intraperitoneally daily from week 8 to 10; Aging + A740003 (AT) Group: rats were injected intraperitoneally with D-galactose (200 mg/kg/day) dissolved in distilled water for 10 weeks [33], and A740003 (19 mg/kg/day) [30] dissolved in a vehicle including 40% distilled water, 50% normal saline, and 10% dimethyl sulfoxide was administered intraperitoneally daily from week 8 to 10. Upon completion of the experiment, rats were fasted for twelve hours and then euthanized with gradually increasing concentrations of carbon dioxide. Blood samples were centrifuged for 20 min at 3500 rpm, and serum samples were preserved at −80 °C. The hearts were then directly removed, immersed in normal saline, and weighed to calculate the heart weight (HW) to body weight (BW) ratio. A section of the left ventricle tissue was frozen in liquid nitrogen and kept at −80 °C for protein and RNA extraction. Another section of the left ventricle tissue was preserved in 10% formalin for histological analysis and immunohistochemistry.

### 4.4. Histological Examination of Heart Tissues

Transverse (cross-sectional) samples of heart tissue were immersed overnight in 10% formalin, then dehydrated with ethanol and processed into paraffin. Heart tissue samples were sectioned at 5 μm thickness and examined for morphology using hematoxylin and eosin (H&E) staining. H staining (blue) showed nuclei, whereas E staining (pink) showed cytoplasm and connective tissue. The photomicrographs were captured using a 400× magnification optical microscope (Nikon, Tokyo, Japan).

### 4.5. Detection of Senescence-Associated Galactosidase Activity

Cellular senescence was determined by measuring SA-β-Gal activity. Briefly, heart tissues were sectioned at 5 μm thickness, and incubated with SA-β-Gal stain for 24 h in 1 mg/mL of X-Gal at pH 6.0 and 37 °C. After incubation, the samples were examined using an optical microscope (Nikon, Tokyo, Japan). Senescent cells were distinguished by blue cytoplasmic staining and counted using ImageJ software. The ratio of senescent cells to total cells was used to determine the percentage of SA-β-Gal-positive cells.

### 4.6. Detection of a Systemic Inflammation

CRP levels, a biomarker of systemic inflammation, were measured in serum using a rat-specific ELISA kit, as directed by the manufacturer.

### 4.7. Detection of Cardiac Inflammaging

Cardiac inflammaging biomarkers, including pro-inflammatory cytokines (TNF-α and IL-6), and NF-κBp105 levels were detected in cardiac tissue using a rat-specific ELISA kit. To detect target protein levels, 100 mg of rat ventricular heart tissue were homogenized from at least six different rats per group, as instructed by the manufacturer. Levels of pro-inflammatory cytokines and NF-κBp105 were measured, and standard curves were created following the manufacturer’s instructions. The colorimetric data were analyzed using a BioTek Microplate Reader (BioTek Instruments, Winooski, VT, USA) at a wavelength of 450 nm. The total concentrations of IL-6 (pg/mL), TNF-α (pg/mL), and NF-κBp105 (pg/mL) were evaluated using linear regression based on standard concentrations.

### 4.8. Immunohistochemistry of Heart Tissues

Immunohistochemistry was used to assess the cardiac expression of p16^Ink4a^, P2X7R, and phospho-NF-κBp105, as described previously [48]. Briefly, heart tissues were deparaffinized in xylene and rehydrated with ethanol. The antigen was retrieved by heating in citrate buffer (10 mM, pH 6.0) for 20 min and then incubating for 20 min with 3% hydrogen peroxide to inhibit the activity of endogenous peroxidase. The sections were then rinsed with phosphate-buffered saline and subjected to overnight incubation with primary antibodies against p^16Ink4a^ (1:100), P2X7R (1:100), and phospho-NF-κBp105 (1:100). Immunoreactivity was visualized with 3,3′-diaminobenzidine, then stained with Mayer’s hematoxylin, and examined under a light microscope. Quantification of the staining-positive area was performed using ImageJ software by a blinded investigator.

### 4.9. Real-Time Polymerase Chain Reaction (RT-PCR) Analysis

The mRNA expression of *p16^Ink4a^*, *p21^Cip/Waf^*, *TNF-α*, *IL-6*, *P2X7R*, *NF-κBp65*, and *IκBα* genes was analyzed in ventricular cardiac tissue. Total RNA was extracted from ventricular cardiac tissues using the RNAbler cells and tissue RNA extraction kit (Haven Scientific, Makkah, Saudi Arabia) in accordance with the manufacturer’s instructions. Complementary DNA (cDNA) was generated from total RNA using the RT-Ace First Strand cDNA Synthesis Kit (Haven Scientific, Saudi Arabia) following the manufacturer’s protocol. The target genes were amplified using quantitative RT-PCR with EverGreen Universal Real-Time PCR Master Mix (Haven Scientific, Saudi Arabia). Forward and reverse primers were provided from Haven Scientific and were as follows: p16^INK4a^ forward, 5′-TGCAGATAGACTAGCCAGGGGA-3′ and reverse, 5′-CTTCCAGCAGTGCCCGCA-3′ [49]; p21^Cip/Waf^ forward, 5′-CCGAGAACGGTGGAACTTTGAC-3′ and reverse, 5′-GAACACGCTCCCAGACGTAGTTG-3′ [49]; TNF-α forward, 5′-ACTGAA CTTCGGGGTGATCG-3′ and reverse, 5′-GCTTGGTGGTTTGCTACGAC-3′ [50]; IL-6 forward, 5′-CATTCTGTCTCGAGCCCACC-3′ and reverse, 5′-GCTGGAAGTCTCTTGCGGAG-3′ [51]; P2X7R forward, 5′-CGAAGTTAGTACACGGCATCTT-3′ and reverse, 5′-CTTGGCCTTCTGACTTGAGATAA-3′ [52]; NF-κBp65 forward, 5′-CGCGGGGACTATGACTTGAA-3′ and reverse, 5′-AGTTCCGGTTTACTCGGCAG-3′ [50]; IκBα forward, 5′-ATGGAAGTGATTGGTCAGGTGA-3′ and reverse, 5′-AGGCAAGATGGAGAGGGGTATT-3′ [53]; GAPDH forward, 5′-AGTGCCAGCCTCGTCTCATA-3′ and reverse, 5′-GATGGTGATGGGTTTCCCGT-3′ [52]. mRNA expression levels were normalized using GAPDH as an internal control. The relative expression of p16^Ink4a^, p21^Cip/Waf^, TNF-α, IL-6, P2X7R, NF-κBp65, and IκBα was quantified as fold change using the 2^−ΔΔCt^ method.

### 4.10. Statistical Analysis

Data were analyzed with GraphPad Prism Software version 10.2.1 and expressed as mean ± standard error of the mean. One-way analysis of variance (ANOVA) and Dunnett’s multiple comparisons test were used to determine group differences at a significance level of *p* < 0.05.

## 5. Conclusions

P2X7Rs mediate aging-related cardiac cellular senescence by regulating the inflammaging pathway. The present study shows that the P2X7R antagonist A740003 can alleviate certain forms of senescent cardiomyocytes, especially those caused by chronic low-grade inflammation associated with aging, and demonstrates that P2X7R antagonist A740003 provides anti-inflammaging effects in cardiac tissue by inhibiting the NF-κB pathway. These findings contribute to our understanding of the molecular mechanisms underlying age-related cardiac inflammation, highlighting P2X7R as a promising target for the treatment and prevention of aging-related cardiovascular diseases. To validate the beneficial effects of P2X7R antagonists in aging-related cardiac injury, more research is needed to elucidate their effects on other features associated with cardiac aging, such as mitochondrial dysfunction, diminished autophagy, myocardial fibrosis, and deteriorating cardiac function.

## Figures and Tables

**Table 1:** Effects of A740003 treatment on body weight, heart weight, and heart weight-to-body weight ratio in D-galactose-induced cardiac aging.

Groups	Body Weight (g)	Heart Weight (g)	HW/BW%
Normal Control	392.7 ± 36.47	1.23 ± 0.12	0.31 ± 0.01
Aging Control	352.3 ± 19.32	1.03 ± 0.05	0.29 ± 0.006
Aging + Drug Vehicle	370.2 ± 13.34	1.20 ± 0.05	0.33 ± 0.02
Aging + A740003	337.8 ± 17.62	1.11 ± 0.08	0.33 ± 0.01

## Data Availability

The original contributions presented in this study are included in the article. Further inquiries can be directed to the corresponding author.

## Abbreviations

The following abbreviations are used in this manuscript:

AC	Aging control
ANOVA	Analysis of variance
AV	Aging + Drug Vehicle
AT	Aging + A740003
cDNA	Complementary DNA
CRP	C-reactive protein
ELISA	Enzyme-linked immunosorbent assay
GAPDH	Glyceraldehyde 3-phosphate dehydrogenase
H&E	Hematoxylin and eosin
IκB	Inhibitor of κB
IL-6	Interleukin-6
NC	Normal control
NF-κB	Nuclear factor-kappa B
P2X7R	Purinergic P2X7 receptor
RT-PCR	Real-Time Polymerase Chain Reaction
SASP	Senescence-associated secretory phenotype
SA-β-Gal	Senescence-associated β-galactosidase
TNF-α	Tumor necrosis factor-alpha
